# In Situ Formation of Highly Durable Subnanometer Platinum Particle Electrocatalysts for Polymer Electrolyte Fuel Cells

**DOI:** 10.1021/acsomega.4c02723

**Published:** 2024-06-11

**Authors:** Hiroshi Yano, Kouta Iwasaki

**Affiliations:** New Field Pioneering Division, Toyota Boshoku Corp., 1-1, Toyoda-cho, Kariya, Aichi 448-8651, Japan

## Abstract

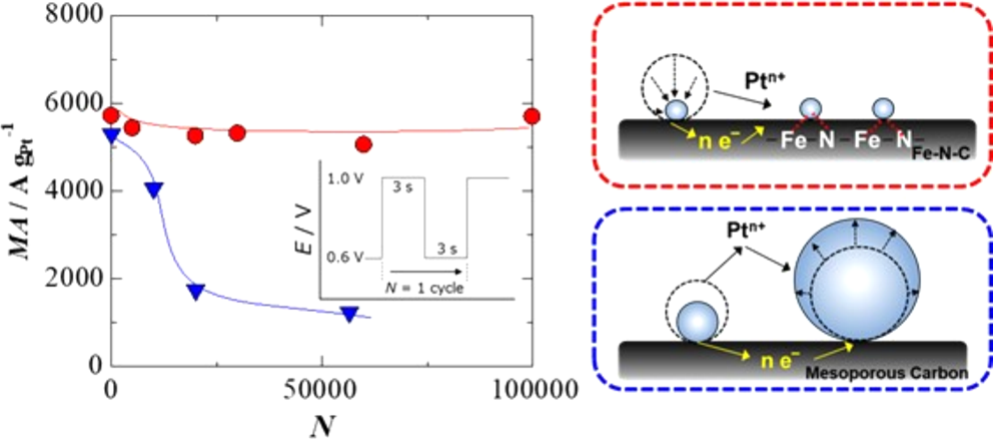

The durability of Pt nanoparticle catalysts is currently the most important factor limiting the widespread use of polymer electrolyte fuel cells (PEFCs). Specifically, the Pt nanoparticles in standard carbon black-supported Pt nanoparticle (Pt/CB) catalysts repeatedly aggregate on the CB surfaces during PEFC operation, thus, reducing the performance of the cell. Therefore, PEFCs must contain large quantities of Pt to maintain sufficient service lifetimes. This is the main factor hindering the reduction of the cost of PEFCs. The present research demonstrates that ultrafine Pt particles (Pt_subnanoes_) having diameters of approximately 0.5 nm can be formed in situ from a platinum chloride complex (PtCl_*n*_) on a carbon-based material doped with Fe and N via the dissolution and reprecipitation of Pt in the PtCl_*n*_ during potential cycling in a 0.1 M HClO_4_ solution. The Pt_subnanoes_ are immobilized by both Fe and N in the support material. The mass-based catalytic activity of this material during the oxygen reduction reaction is eight times higher than that of a standard Pt/CB catalyst and is maintained even after 100,000 potential step cycles (0.6 ↔ 1.0 V). The present results provide guidelines for the development of highly durable yet active membrane electrode assemblies that minimize the use of Pt.

## 1 Introduction

Polymer electrolyte fuel cells (PEFCs), which are expected to serve as the next generation of clean energy supply systems, use a catalyst consisting of highly dispersed nanometer-sized platinum particles supported on a high surface area carbon black-based material (Pt/CB). Pt is the most viable catalyst for the oxygen reduction reaction (ORR) at the cathode. Even so, a large amount of this metal is required to maintain sufficient power output due to the very high overvoltage of this metal and its susceptibility to degradation in standard PEFC operating environments. Therefore, to promote the widespread use of fuel cells, it will be necessary to improve the durability of Pt catalysts and reduce the quantity of this metal that is needed. As an example, the current goal for PEFCs intended for use in fuel cell vehicles (FCVs) is to obtain a Pt consumption of 0.07 g_Pt_ kW^–1^ by 2040. This value represents a reduction from the current Pt consumption per FCV of 20 g_Pt_ to approximately 1 g_Pt_ and must be accomplished without any reduction in performance.^1^

Traditionally, the activity per unit mass of platinum (mass activity, MA) was used as the standard for determining catalyst performance. The MA for the ORR is defined as
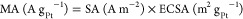
1where SA and ECSA are the specific activity and the electrochemically active surface area per mass of Pt, respectively. The SA value is greatly affected by the specific crystalline orientation of Pt and the proportion of index planes, with a particular crystallographic orientation on the Pt surface increases as the Pt particle diameter (*d*) decreases.^2,3^ Because ECSA is inversely proportional to *d*, the use of Pt nanoparticles can simultaneously increase both SA and ECSA, thus significantly improving MA. This effect of particle size has been known for some time now. As an example, Gasteiger et al. demonstrated that SA is strongly dependent on *d* over the range from 2 nm particles to bulk material.^4^

FCVs typically operate at 60–90 °C and the Pt/CB catalyst is frequently exposed to variations in load and/or start/stop conditions. In this harsh environment, it is challenging to maintain the size, shape, and/or specific crystalline orientation of especially small Pt nanoparticles.^5^ Therefore, it is necessary to prepare relatively large Pt nanoparticles (ca. 5–10 nm)^6,7^ in order to increase the durability of the material. Unfortunately, these larger particles do not allow the amount of Pt being used to be reduced because the MA decreases with increasing Pt particle size. Many researchers have struggled with this dilemma for some time now.

The recent development of highly functional catalysts has been based on exploiting the unusual properties of certain carbon materials to increase the durability of Pt nanoparticles. Carbon nanotubes,^8^ mesoporous carbon,^9,10^ and Fe and/or N-doped carbon^11−17^ are typical examples. It has been confirmed that the unique defect sites and pore structures of these materials can modify the catalyst durability, although not to the point that these changes can be considered to be breakthroughs. Ultimately, researchers in this field have not yet found an optimal approach to enhance the durability of Pt nanoparticles.

The present work demonstrates a novel functional catalyst consisting of subnanometer-sized Pt particles (Pt_subnano_) and Fe- and N-doped carbon (Fe–N–C). The Pt_subnano_ particles were prepared in situ from Pt chloride complexes (PtCl_*n*_), after these complexes were fixed on Fe–N–C, by potential cycling in acidic solutions. Hereinafter, the catalyst obtained by this process will be referred to as PtCl_*n*_/Fe–N–C and Pt_subnano_/Fe–N–C before and after potential step cycling (0.6 ↔ 1.0 V), respectively. The Pt_subnano_/Fe–N–C catalyst showed superior durability based on the immobilization of the Pt particles on both N and Fe atoms in the Fe–N–C.

## 2 Experimental Section

### 2.1 Preparation of PtCl_*n*_/Fe–N–C

The PtCl_*n*_/Fe–N–C catalysts were prepared by mixing 60 mg of powdered hydrogen hexachloroplatinate(IV) hexahydrate (H_2_PtCl_6_·6H_2_O, Kanto Chemical Co., Japan) and 50 mg of supplied Fe and N-doped carbon (Fe–N–C), which was prepared by hard templating method with fumed silica,^11,16^ in ethanol (1 mL) in a mortar. The Fe and N concentrations in the carbon were approximately 0.5 and 5 wt %, respectively. Mesoporous carbon (MPC, CNovel) and graphitized carbon black (GCB, EA, Tanaka Kikinzoku Kogyo)^18^ powders were also used as reference carbon-based support materials. Each mixture was stirred while the materials were warmed using a heat gun until the ethanol was almost completely evaporated. The powders were then completely dried by heating at 60 °C for 30 min under vacuum and subsequently heat-treated under an Ar atmosphere at 200 °C for 2 h in a furnace.

### 2.2 Electrochemical Assessments

All electrochemical data were obtained using a rotating ring disk electrode (RRDE) system (RRDE-3A, ALS Co., Ltd.) with a gastight water-jacketed Pyrex glass cell. The working electrodes consisted of a thin layer of the PtCl_*n*_/Fe–N–C, PtCl_*n*_/MPC, or PtCl_*n*_/GCB catalyst uniformly dispersed on a glassy carbon disk substrate having a diameter of 4 mm. The catalyst suspension was prepared by mixing 2 mg of catalyst powders with 2 mL of ethanol (*C*_cat._ = ca. 1.0 g L^–1^), followed by ultrasonication for 10 s. Then, a catalyst ink of ca. 30 μL was pipetted onto a glassy carbon disk. After that, 2 μL of 0.2 wt % Nafion solution was dropped onto the catalyst layer and then dried in air at 60 °C for 30 min. The preparation conditions of the catalysts suspension and resulting the amount of Pt and carbon on glassy carbon disk are summarized in Table 1. Standard commercial Pt/CB (TEC10E50E, TKK) was also used as a reference catalyst. The properties of commercial Pt/CB are shown in Table S1 in the Supporting Information (SI). To check the reproducibility of the electrochemical properties of PtCl_*n*_/Fe–N–C, it was prepared again using the same recipe as above (denoted *PtCl_*n*_/Fe–N–C). Details of the properties are shown in Figure S1 in the SI. A 0.1 M HClO_4_ electrolyte solution was prepared from Suprapur grade HClO_4_ and Millipore-Q water (Millipore Japan Co., Ltd.).

**Table 1:** Typical Properties of the Prepared Catalyst and Preparation Condition of Catalysts Suspensions

	specific surface area of carbon, SSA (m^2^ g^–1^)	Pt loaded, *y*^a^ (wt %)	particle size, *d*^b^ (nm)	interparticle distance, *X*_Pt–Pt_^c^ (nm)	amount of catalyst in the ink suspension, *C*_cat._ (g L^–1^)	amount of the pipetting the suspension, *V*_ink_ (μL)	amount of Pt attached in the catalyst layer, *m*_Pt_ (μg cm^–2^)	amount of carbon attached in the catalyst layer, *m*_c_ (μg cm^–2^)
PtCl_*n*_/Fe–N–C	560	24.1	1.4 ± 0.2	7.7	1.0	29	55.6	175.1
PtCl_*n*_/GCB	150	12.6	1.4 ± 0.2	6.0	1.1	40	38.1	264.3
PtCl_*n*_/MPC	460	27.4	1.4 ± 0.2	6.4	1.1	26	62.3	165.2

a Pt weight percent in PtCl_*n*_/Fe–N–C, PtCl_*n*_/GCB, PtCl_*n*_/MPC, and Pt/CB catalysts estimated by weight loss, using thermogravimetry (TG).

b Average particle size and standard deviations based on the TEM observation.

c The average interparticle distance *X*_Pt–Pt_ was calculated by the following equation: *X*_Pt–Pt_ = {πσ*d*^3^*S*_c_(100 – *y*)/3√3 × *y*}^1/2^ where σ is the density of Pt (g nm^–3^), *d* is the average particle size of Pt (nm), *S*_c_ is the specific surface area of carbon support (nm^2^ g^–1^), and *y* is the Pt loaded on the support.^22^

Accelerated stress testing (AST) of the catalysts was performed according to the procedure shown in Figure S2 in the SI. In the first step of this process, conventional cyclic voltammetry (CV) data were acquired in a 0.1 M HClO_4_ solution deaerated with Ar (G1-grade, 99.9999%) at 30 °C to estimate the ECSA value. This value was calculated from the electric charge associated with the hydrogen desorption wave, Δ*Q*_H_, in each CV plot (as shown in Figure S3), assuming Δ*Q*_H_ = 210 μC cm^–2^ for smooth polycrystalline Pt.^19,20^ Subsequently, the ORR activity and the formation of H_2_O_2_ (a byproduct of the ORR) at the Nafion-coated catalyst electrode in a 0.1 M HClO_4_ solution saturated with O_2_ (G2-grade, 99.999%) were investigated using the RRDE technique. Hydrodynamic voltammetry for the ORR were recorded from 0.3 to 1.0 V at a scan rate of 10 mV s^–1^. During this stage, the potential of the ring electrode was set to 1.2 V to allow H_2_O_2_ emitted from the disk electrode to be detected. In the third step, each electrode was subjected to accelerated degradation in a 0.1 M HClO_4_ solution deaerated with Ar according to the standard potential step protocol recommended by the Fuel Cell Commercialization Conference of Japan.^21^ After a specific number of potential step cycles (*N*), the procedure from step 1 was repeated.

### 2.3 Characterization of the Catalysts

Catalyst powder specimens were observed by transmission electron microscopy (TEM, Hitachi H-9500, acceleration voltage = 200 kV; JEOL JEM-F200, acceleration voltage = 200 kV) and scanning transmission electron microscopy (STEM, Hitachi HD-2700, acceleration voltage = 200 kV) before and after AST to determine the sizes and size distributions of the Pt particles. The structures of these particles were analyzed by X-ray diffraction (XRD, Rigaku Ultima IV), electron diffraction (ED), and energy dispersive X-ray spectroscopy (EDX, EDAX r-TEM). X-ray photoelectron spectroscopy (XPS) was used to determine the surface electronic states of the catalyst powders. These data were collected using a PHI ESCA-5800 spectrometer with an Al Kα monochromatic source operating at 300 W with a photoelectron takeoff angle of 45°, corresponding to a measurement depth of 4 nm. All samples were reduced under high purity H_2_ (G1-grade, 99.9999%) in a furnace tube at room temperature for 2 h prior to analysis. X-ray absorption fine structure (XAFS) spectra (L-edge for platinum and K-edge for iron) were generated on the BL14B2 beamline at the SPring-8 facility by employing a Si(111) monochromator. Prior to analysis, the samples were reduced using the same procedure as described above. After reduction, the samples were ground in a mortar and pressed into pellets each with a 10 mm diameter and 1 mm thickness in a glovebox filled with Ar. The entire analytical process was carried out without exposing the samples to air.

## 3 Results and Discussion

### 3.1 Characterization of Pristine Catalysts

**Figure 1:**
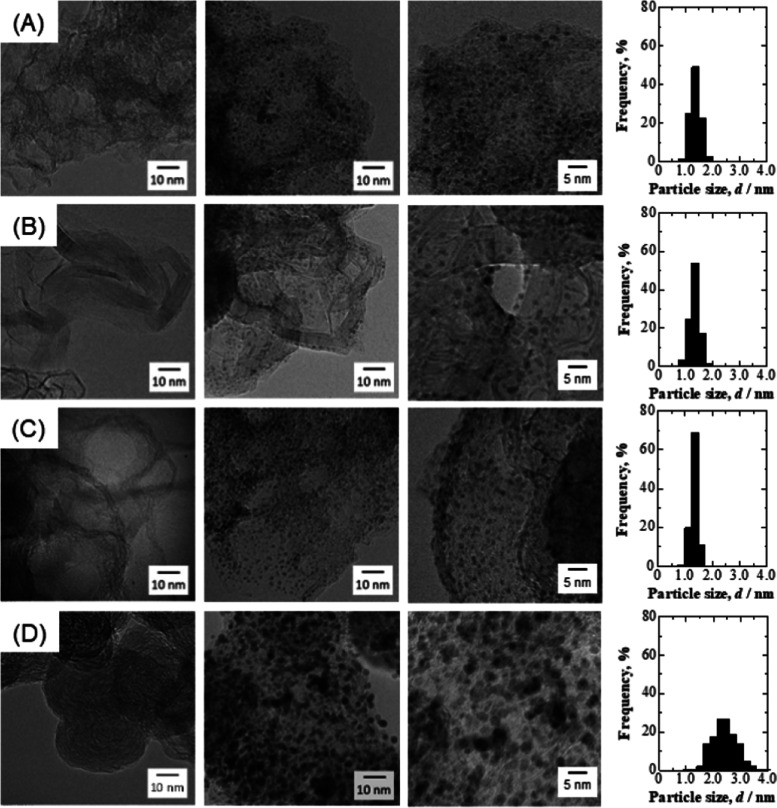
TEM images [left: carbon materials used as supports; (A) iron–nitrogen doped carbon (Fe–N–C), (B) graphitized carbon black (GCB), (C) mesoporous carbon (MPC), and (D) carbon black (CB), middle and right: low- and high- magnification of highly dispersed PtCl_*n*_ on each carbon; PtCl_*n*_/Fe–N–C, PtCl_*n*_/GCB, PtCl_*n*_/MPC, and Pt/CB (commercial)]. Particle size distribution histograms for each catalyst powder. The histograms were obtained among 300 particles in the several images.

Figure 1 shows TEM images of the four carbon materials used as catalyst supports and of PtCl_*n*_/Fe–N–C, PtCl_*n*_/GCB, PtCl_*n*_/MPC, and commercial Pt/CB specimens together with particle size distribution histograms. In the TEM images, the PtCl_*n*_ appears as black particulates, as do spherical Pt nanoparticles in the commercial Pt/CB. Therefore, for convenience, the particle size distributions of the PtCl_*n*_/Fe–N–C, PtCl_*n*_/GCB, and PtCl_*n*_/MPC were generated assuming spherical particles. The average particle size, *d*_TEM_, and standard deviation, σ_d_, determined for these three materials were 1.4 ± 0.2, 1.4 ± 0.2, and 1.4 ± 0.1 nm, respectively. The size distributions of these three PtCl_*n*_ catalysts were thus relatively narrow compared with the commercial Pt/CB (*d*_TEM_ = 2.5 ± 0.4 nm). Typical properties of the catalyst powders are summarized in Table 1. The interparticle distance (*X*_Pt–Pt_) was calculated from the specific surface area of the carbon support, the metal loading, and the Pt particle sizes,^22^ and all three catalysts showed essentially equivalent values. This parameter is very important, with regard to the catalytic activity. This is because the ORR is inefficient due to the diffusion fields of O_2_ on the catalyst overlapping when the particles are in close proximity to one another.^23^ Thus, the data confirm that three different catalysts having the same particle sizes, size distributions, and interparticle distances were synthesized. The particle size and size distribution of *PtCl_*n*_/Fe–N–C were also almost identical to that of PtCl_*n*_/Fe–N–C (see in Figure S1), confirming the reproducibility of the synthesis.

**Figure 2:**
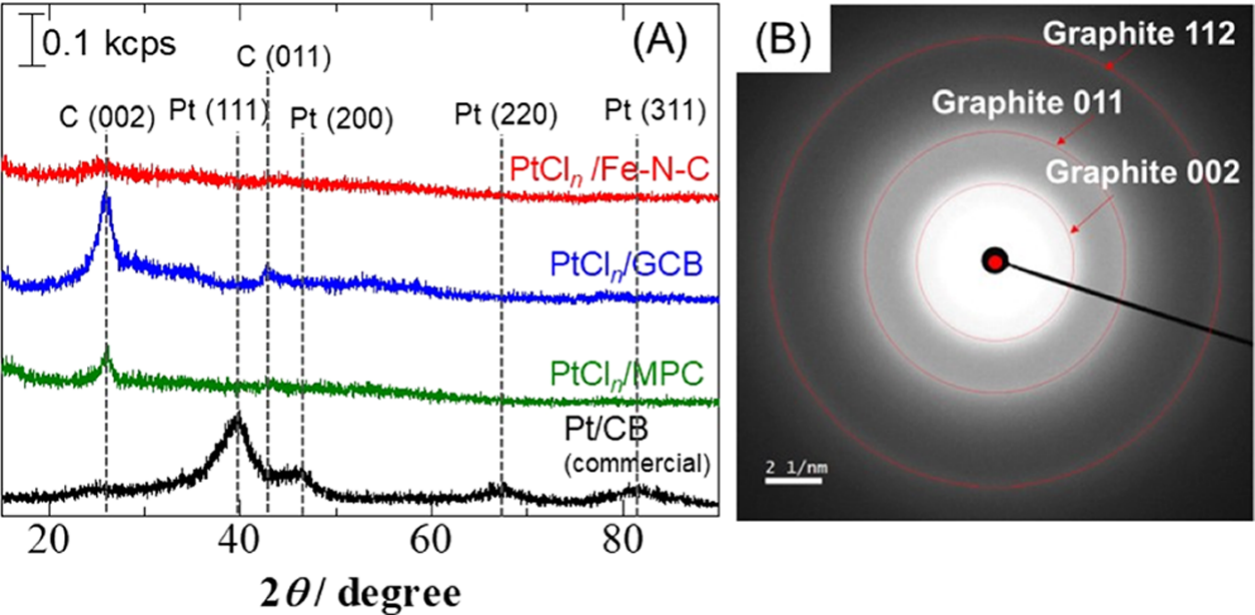
(A) XRD patterns of pristine PtCl_*n*_/Fe–N–C, PtCl_*n*_/GCB, PtCl_*n*_/MPC, and Pt/CB powders, and (B) the ED pattern of pristine PtCl_*n*_/Fe–N–C.

XRD patterns of pristine PtCl_*n*_/Fe–N–C, PtCl_*n*_/GCB, PtCl_*n*_/MPC, and Pt/CB specimens and the ED pattern of the pristine PtCl_*n*_/Fe–N–C are presented in Figure 2A,B, respectively. The diffraction peaks obtained from the Pt/CB were assigned to the face-centered cubic (fcc) phase of Pt. In contrast, only a few of the peaks produced by the PtCl_*n*_/Fe–N–C, PtCl_*n*_/GCB, and PtCl_*n*_/MPC were attributable to Pt. Similarly, the ED pattern did not exhibit Pt rings. It is assumed from these results that the PtCl_*n*_ particles were extremely small (<2 nm) and had a low degree of crystallinity, but the presence of Pt is unquestionable from the XPS (Figure 5) and XAFS (Figure 6) measurements discussed in Section 3.4.

### 3.2 AST of Prepared Catalysts

**Figure 3:**
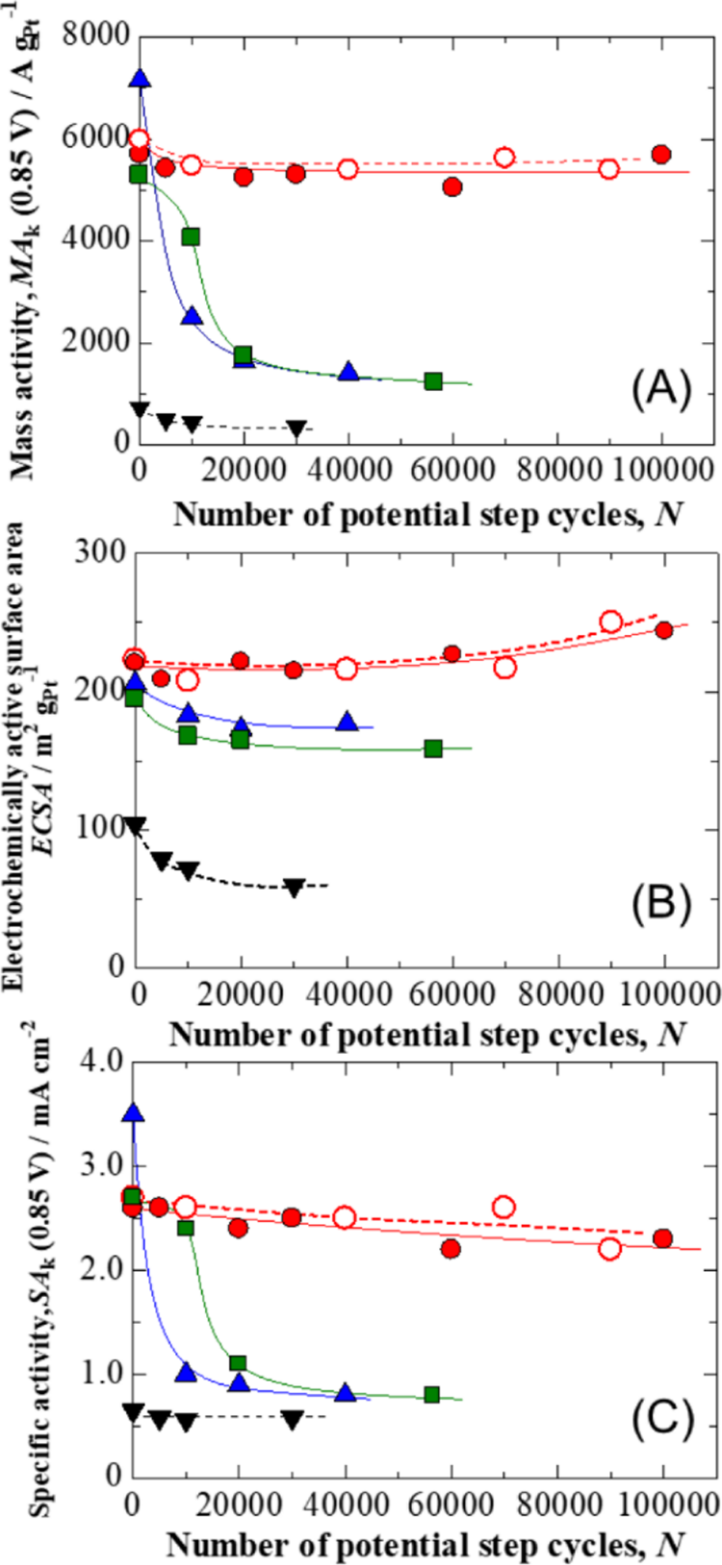
Change in (A) kinetically controlled mass activity, MA_k_, (B) electrochemically active area, ECSA, and (C) kinetically controlled area-specific activity, SA_k_, at Nafion-coated PtCl_*n*_/Fe–N–C (red circle solid), *PtCl_*n*_/Fe–N–C (red circle open), PtCl_*n*_/GCB (blue triangle up solid), PtCl_*n*_/MPC (green box solid), and Pt/CB (▼) electrodes as a function of the number of potential step cycles, *N*. The values of SA_k_ and MA_k_ were evaluated at 0.85 V vs RHE.

The kinetically controlled specific activity, SA_k_, and mass activity, MA_k_, were obtained from hydrodynamic voltammetries based on the ORR. As an example, the hydrodynamic voltammetries generated using a Nafion-coated PtCl_*n*_/Fe–N–C, PtCl_*n*_/GCB, PtCl_*n*_/MPC, and Pt/CB disk electrodes in an O_2_-saturated 0.1 M HClO_4_ solution at 30 °C and the simultaneously acquired H_2_O_2_ oxidation currents at the Pt ring electrodes are shown in Figure S4. The kinetically controlled current, *I*_k_, at 0.85 V was calculated using the Koutecky–Levich equation
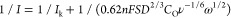
2where *n* is the number of electrons transferred, *F* is the Faraday constant, *S* is the effective projected area of the Pt catalyst, *D* is the diffusion coefficient of O_2_, *C*_O_ is the O_2_ concentration, ν is the viscosity of the electrolyte, and ω is the angular velocity. The ORR activity was evaluated at 0.85 to 0.87 V to avoid the formation of oxide species on the Pt surface at high potential (*E* > 0.9 V)^24^ and to evaluate in the appropriate kinetically controlled range. The *I*^–1^ vs ω^1/2^ plots (that is, the Koutecky–Levich plots) for the ORR at 0.85 V on the Nafion-coated PtCl_*n*_/Fe–N–C, PtCl_*n*_/GCB, PtCl_*n*_/MPC, and Pt/CB electrodes are shown in Figure S5. A linear relationship with a constant slope was obtained for each of the electrodes. By extrapolation of the plots to ω^1/2^ = 0 (meaning an infinite mass transfer rate), values of *I*_k_ could be calculated. In addition, MA_k_ values were determined by dividing *I*_k_ by the amount of Pt initially loaded on the disk electrode, and SA_k_ values were calculated by dividing the ECSA by the MA_k_. Figure 3 plots MA_k_, ECSA, and SA_k_ for the Nafion-coated PtCl_*n*_/Fe–N–C, PtCl_*n*_/GCB, PtCl_*n*_/MPC, and Pt/CB electrodes at 0.85 V as functions of the number of potential step cycles (*N*) during the AST. The MA_k_ values for the PtCl_*n*_/GCB, PtCl_*n*_/MPC, and Pt/CB electrodes are seen to have decreased rapidly with increase in *N*. Surprisingly, the MA_k_ associated with the PtCl_*n*_/Fe–N–C electrode was on the order of 17 times higher than that of the Pt/CB electrode at *N* = 30,000 and was maintained without any decrease at *N* = 100,000. Therefore, we focused on the changes in ECSA and SA_k_ values as a means of assessing the high durability of the PtCl_*n*_/Fe–N–C.

The initial ECSA values (at *N* = 0) of 221 m^2^ g^–1^ (PtCl_*n*_/Fe–N–C), 206 m^2^ g^–1^ (PtCl_*n*_/GCB), 195 m^2^ g^–1^ (PtCl_*n*_/MPC), and 104 m^2^ g^–1^ (Pt/CB) are considered reasonable for the specific surface areas calculated from *d*. The initial values of SA_k_ at PtCl_*n*_/GCB and PtCl_*n*_/MPC electrodes were almost the same or higher than those of the PtCl_*n*_/Fe–N–C electrode, indicating that the SA_k_ is independent of the support material. The ECSA and SA_k_ values for the PtCl_*n*_/Fe–N–C and *PtCl_*n*_/Fe–N–C electrodes increased slightly or remained almost constant with increasing *N*, and these changes agreed well. In contrast, both ECSA and SA_k_ decreased with *N* for the PtCl_*n*_/GCB and PtCl_*n*_/MPC electrodes. In particular, both electrodes showed a significant decrease in SA_k_ values, which had a significant impact on the rate of decrease of MA_k_. This effect can possibly be attributed to the disappearance of specific planes of the Pt particles as a consequence of Ostwald ripening.^25^ A similar change in the ORR activity was observed at 0.87 V (Figure S6). Essentially, the changes in the ECSA and SA_k_ values for the PtCl_*n*_/Fe–N–C and *PtCl_*n*_/Fe–N–C were unique compared with the other electrodes. It is unlikely that the Cl atoms in PtCl_*n*_ directly contributed to the uniqueness (that is, the high durability) of the catalyst. The preparation of PtCl_*n*_ and the techniques used to load this material onto the GCB and MPC supports were the same as for the Fe–N–C support. Therefore, the most likely reason for the exceptional durability of the PtCl_*n*_/Fe–N–C and *PtCl_*n*_/Fe–N–C electrodes was likely the interaction between the PtCl_*n*_ particles and the Fe–N–C support. In fact, Xiao et al. previously found that interactions between Fe and Pt enhance ORR activity.^17^ On this basis, the fine structure of the PtCl_*n*_/Fe–N–C before and after AST was assessed using various analytical instruments as a means of explaining the durability of this specimen.

### 3.3 TEM and STEM Observations

**Figure 4:**
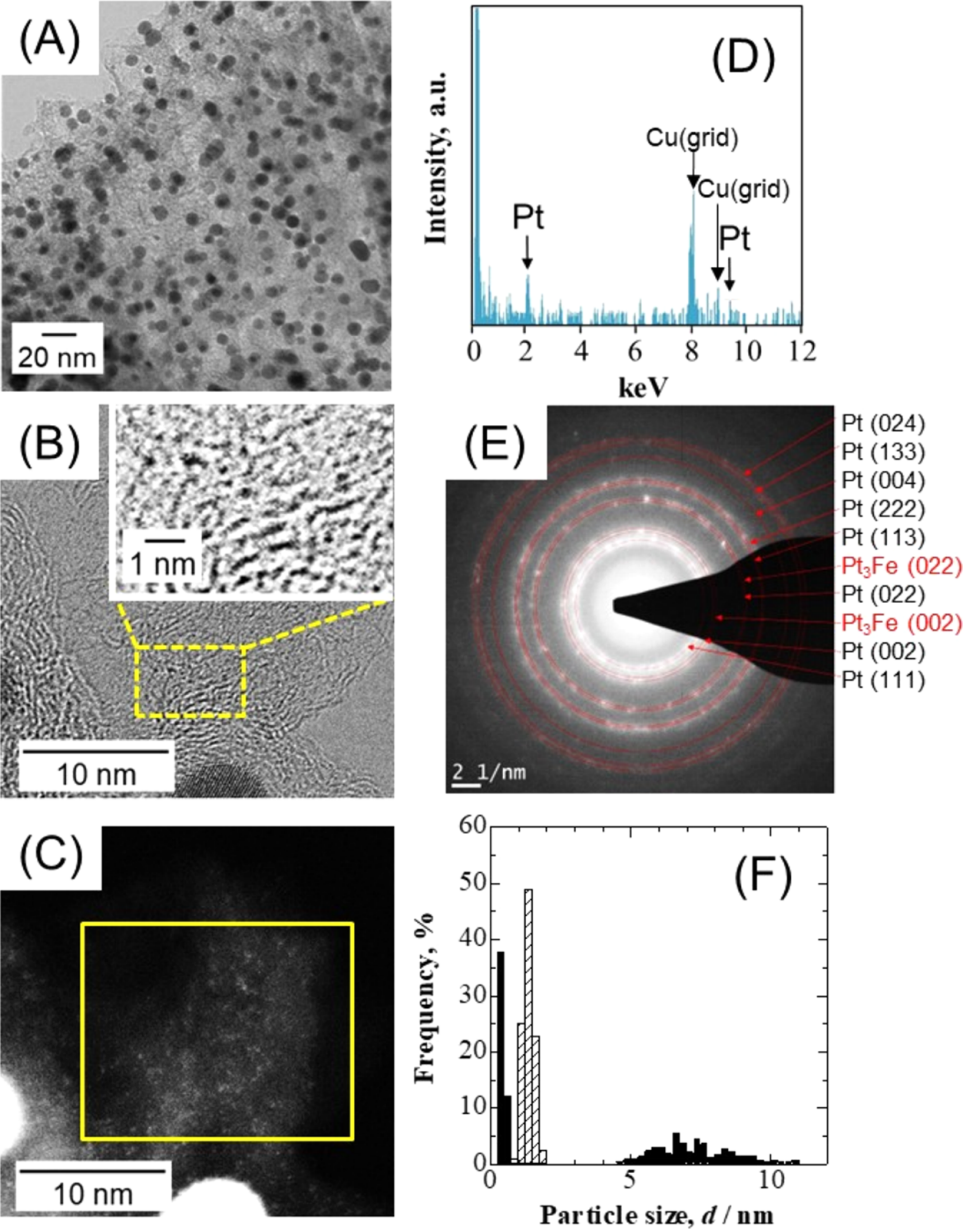
Characterizations of the PtCl_*n*_/Fe–N–C catalyst after AST (Pt_subnano_/Fe–N–C). (A) A TEM image, (B) a high magnification scanning TEM image (inset: ultrahigh magnification of the yellow dotted area), (C) a HAADF-STEM image of the sample in panel (B), (D) EDX spectrum of the yellow square area in panel (C), (E) selected-area electron diffraction pattern, (F) particle size distribution histograms before (hatched bars) and after (black bars) AST.

Figure 4(A) shows a low magnification TEM image of the PtCl_*n*_/Fe–N–C catalyst after AST (*N* = 100,000). The Pt particles were clearly coarsened compared with the pristine powder (Figure 1(A)). The enlarged regions between the coarsened platinum particles seen in Figure 4(A) were observed by STEM. Bright-field and dark-field images of the same area are shown in Figure 4(B,C), respectively. It was found that ultrafine particles of approximately 0.5 nm were present throughout the Fe–N–C support (see the black dots in the ultrahigh magnification image inset in Figure 4(B)). An EDX spectrum is shown in Figure 4(D), obtained from the area denoted by the yellow square in Figure 4(C). This area did not contain coarsened Pt particles; therefore, these particles were evidently made of platinum. Figure 4(E) provides the ED pattern for a selected area in Figure 4(A). These fringe-like patterns were assigned to the (024), (133), (004), (222), (113), (022), (002), and (111) planes of fcc Pt. Patterns of this type were not obtained from the pristine powder (Figure 2(B)). These results indicate that the potential cycling during AST converted PtCl_*n*_ to metallic Pt and also promoted crystallization. Interestingly, fringe-like patterns assigned to Pt_3_Fe alloys (i.e., to (022) and (002) lattice planes) were also identified.

The particle size distribution histograms acquired after AST from two types of Pt particles with average *d* of 0.5 nm (Pt_0.5nm_) and 7 nm (Pt_7nm_) are provided in Figure 4(F). Each distribution was produced by counting 300 particles from multiple images of appropriate resolution and, thus, is not associated with any particular region. The ratio of the number of Pt_0.5nm_ to Pt_7nm_ was calculated from the amount of loaded Pt and the ECSA value after AST (244 m^2^ g^–1^) to be approximately Pt_0.5nm_/Pt_7nm_ = 5000/1 (using Pt specific surface areas as calculated using *d* = 0.5 and 7 nm of 560 and 40 m^2^ g^–1^, respectively). Observations of the state of the catalyst after AST suggested that Pt ions dissolved from the PtCl_*n*_ formed new subnanometer-sized Pt particles (Pt_subnano_) on the Fe–N–C surface during the potential cycling (hereafter, the PtCl_*n*_/Fe–N–C after AST is therefore denoted as Pt_subnano_/Fe–N–C). These ultrafine particles, approximately 0.5 nm in size, greatly increased the catalytic performance while limiting the decrease in the ECSA (Figure 3B).

### 3.4 XPS and XAFS

**Figure 5:**
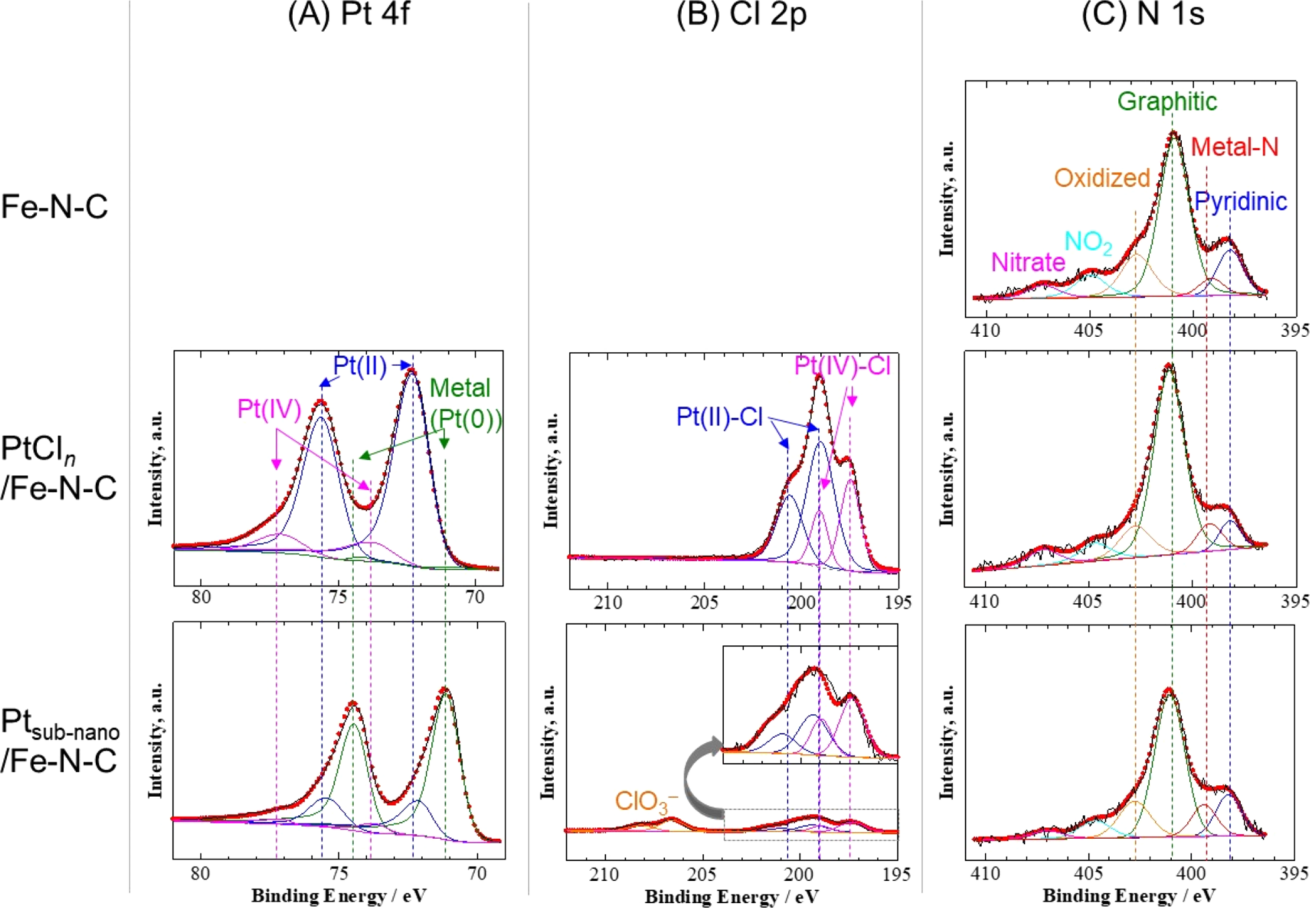
XPS data obtained from the Fe–N–C, PtCl_*n*_/Fe–N–C, and Pt_subnano_/Fe–N–C powders in the (A) Pt 4f, (B) Cl 2p, and (C) N 1s energy regions.

**Figure 6:**
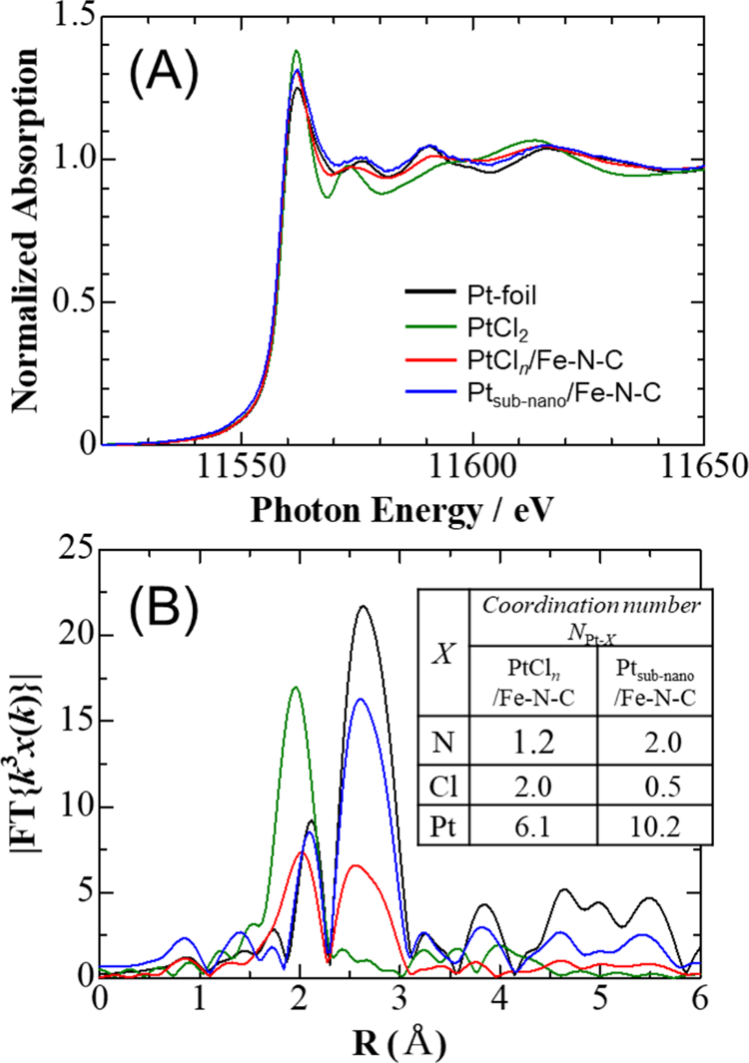
(A) Pt L-edge XANES data and (B) Fourier transforms of normalized *k*_3_ weighted EXAFS spectra for the PtCl_*n*_/Fe–N–C and Pt_subnano_/Fe–N–C powders after *N* = 5000. Data for Pt foil and PtCl_2_ are included as references.

The interactions between the ultrafine Pt particles and Fe–N–C support were examined by using X-ray techniques. First, the chemical states of Pt and Cl atoms in the PtCl_*n*_ and of N atoms in the Fe–N–C support were investigated by XPS and XAFS. Here Pt_subnano_/Fe–N–C was analyzed with samples after *N* = 5000 in order to avoid the information of coarsening Pt as much as possible. The narrow scan XPS spectra and the fitting curves in the Pt 4f, Cl 2p, and N 1s energy regions for Fe–N–C, PtCl_*n*_/Fe–N–C, and Pt_subnano_/Fe–N–C (after *N* = 5000 cycles) are shown in Figure 5. A peak shift is apparent in the Pt 4f spectra (Figure 5(A)) after AST. The peak fitting of these data indicated that Pt was primarily in the form of Pt(II) and Pt(IV)^26^ prior to AST and then was largely reduced to Pt(0) after AST. Fitting of the Cl 2p spectra (Figure 5(B)) demonstrated two different states for Cl with lower (199.2 eV) and higher (197.5 eV) binding energies prior to AST, assigned to Pt(II)–Cl (PtCl_2_) and Pt(IV)–Cl (PtCl_4_), respectively.^27^ Thus, the majority of Pt(II) and Pt(IV) compounds for which peaks appeared in the Pt 4f spectra were likely Pt(II)–Cl (PtCl_2_) and Pt(IV)–Cl (PtCl_4_) derived from H_2_PtCl_6_·6H_2_O. However, considering charge transfer between Pt and N atoms in these materials, the Pt(II) and Pt(IV) peaks could also be attributed to compounds containing Pt–N bonds.^26,28−31^ Fitting of the N 1s spectra (Figure 5(C)) confirmed a peak component attributed to metal–N bonds that increased in intensity in the order of Fe–N–C, PtCl_*n*_/Fe–N–C, and Pt_subnano_/Fe–N–C. In the case of the Fe–N–C, this bond was most likely Fe–N. In contrast, the increase in the intensity of this peak in the case of the PtCl_*n*_/Fe–N–C and Pt_subnano_/Fe–N–C catalysts was likely due to binding with the supported Pt atoms. Therefore, XAFS analyses were used to confirm the presence of Pt–N bonding. Figure 6 shows the Pt-L3 edge X-ray absorption near edge structure (XANES) spectra and radial structure function (RSF) obtained from the Fourier transform of extended X-ray absorption fine structure (EXAFS) spectra for the PtCl_*n*_/Fe–N–C and Pt_subnano_/Fe–N–C. Data for Pt foil and PtCl_2_ are also included for reference, since the white line (WL) of the XANES spectra for Pt compounds with Pt–N bonds appears between those for these materials.^32^ As shown in Figure 6(A), the WLs for both PtCl_*n*_/Fe–N–C and Pt_subnano_/Fe–N–C were located between the Pt foil and PtCl_2_ lines. The RSF (Figure 6(B)) also established the presence of Pt–Pt (with a bond distance of approximately 2.6 Å), Pt–Cl (2.0 Å), and Pt–N bonds (1.8 Å). Following AST, the Pt–Pt (*N*_Pt–Pt_) and Pt–N (*N*_Pt–N_) coordination numbers increased from 6.1 to 10.2 and from 1.2 to 2.0, respectively, while the Pt–Cl coordination number (*N*_Pt–Cl_) decreased from 2.0 to 0.5. From these results, it is concluded that PtCl_*n*_ particles supported on Fe–N–C were immobilized by chemical bonding between the Pt atoms and N atoms of the support. Furthermore, the amount of Pt and N bonding was found to increase with potential cycling in the electrolyte solution.

**Figure 7:**
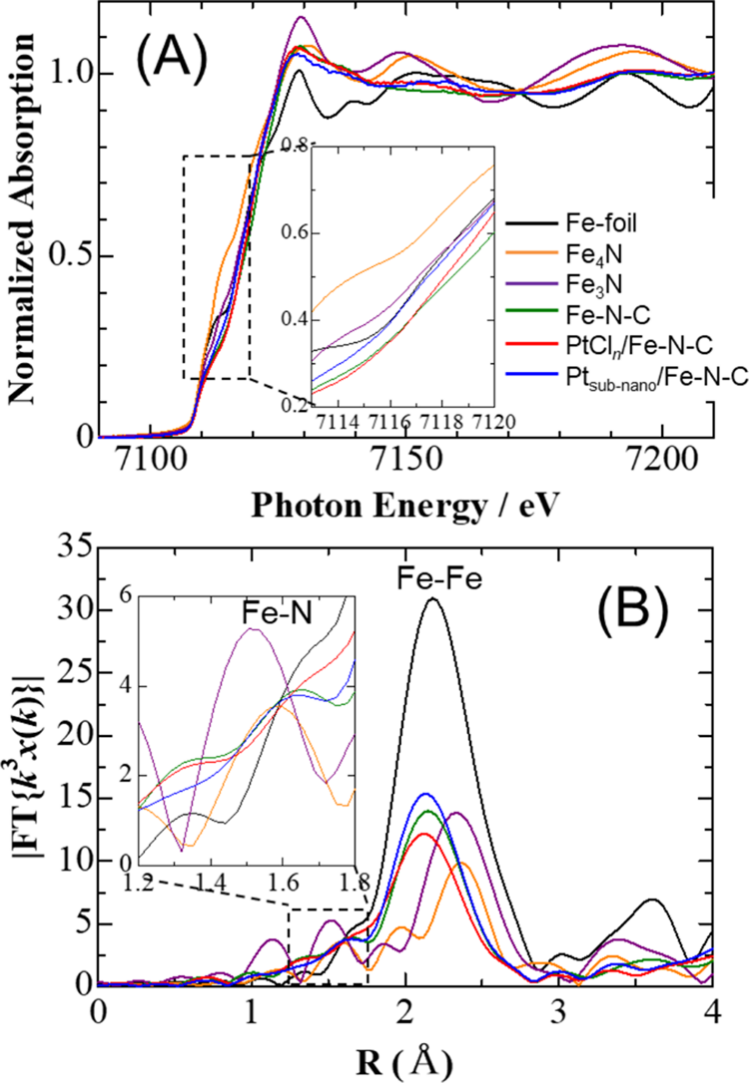
(A) Fe K-edge XANES data and (B) Fourier transforms of normalized *k*_3_ weighted EXAFS spectra for the Fe–N–C, PtCl_*n*_/Fe–N–C, and Pt_subnano_/Fe–N–C powders after *N* = 5000. Fe-foil, Fe_4_N, and Fe_3_N are included as references.

The chemical state of the Fe atoms in the Fe–N–C support was also investigated. Because the Fe 2p XPS spectrum was not strong enough for curve fitting, only an XAFS analysis was performed. Figure 7 presents the Fe–K edge XANES and RSF results for the Fe–N–C, PtCl_*n*_/Fe–N–C, and Pt_subnano_/Fe–N–C powders, along with data for Fe-foil, Fe_4_N, and Fe_3_N used as references. In Figure 7(A), the shoulder at approximately 7117 eV can be ascribed to Fe–N bonds such as Fe–N_4_ and Fe–N_3_.^33^ Hence, the Fe atoms doped into the carbon framework were in close proximity to the N atoms. Fe–N and Fe–Fe peaks can also be seen in the RSF spectra (Figure 7(B)) with bond distances of 1.5 and 2.0 Å, respectively. Because the intensity of the Pt_subnano_/Fe–N–C peaks was slightly increased in comparison with that of PtCl_*n*_/Fe–N–C, it appears that Fe was not leached out even after potential cycling in the acidic solution and that Fe–N–C retained its original structure.

### 3.5 High Durability Mechanism

**Figure 8:**
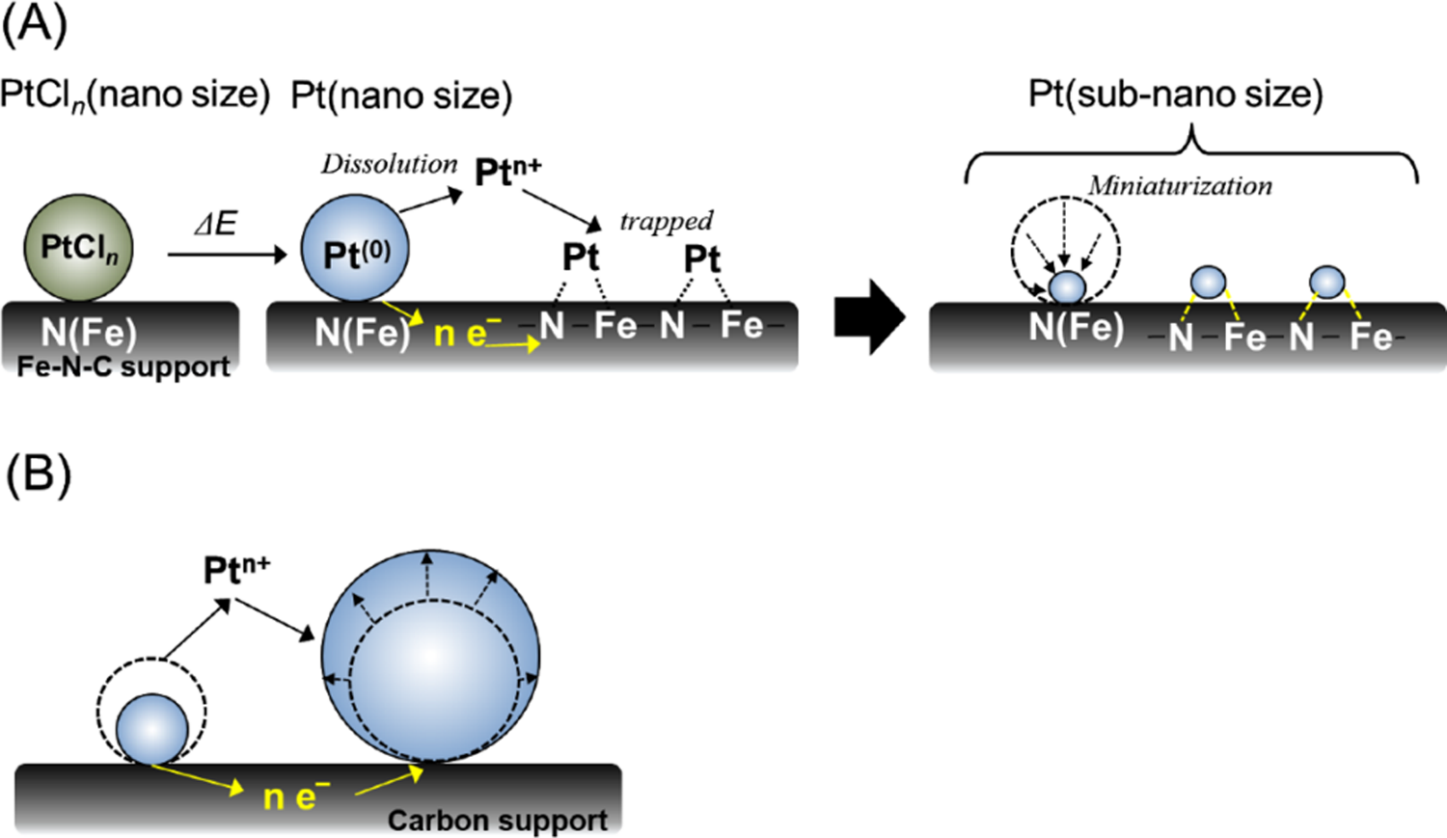
Diagram showing the formation of (A) subnanometer-sized Pt particles on the Fe–N–C support and (B) large Pt particles (coarsening).

As shown in Figure 3, our catalysts show high initial SA_k_ for the ORR. This is because the area occupied by highly active sites relative to the ORR depends on their size.^34−36^ From research on well-defined single crystal Pt electrodes, it is known that ORR activity increases in the order of (111) ≪ (100) < (110).^36^ The particle size with an optimum ratio of (110) planes is considered to be about 1.4 nm, assuming the cuboctahedral model. It is thought that this activity can be maintained due to the interaction of Pt with doped Fe and N atoms. A diagram showing the process by which durable subnanometer-sized Pt particles were obtained from the PtCl_*n*_/Fe–N–C electrode is provided in Figure 8(A). In this mechanism, after PtCl_*n*_ particles are dispersed on the Fe–N–C support, some of the Pt atoms are bound to N and/or Fe atoms on the Fe–N–C such that the PtCl_*n*_ particles are immobilized. When PtCl_*n*_ particles are subjected to potential cycling in an acidic solution to release Pt ions, these ions are trapped by neighboring Fe and N atoms and grow to subnanometer size as new Pt particles (Pt_subnano_). During this process, some Cl atoms dissolve, and a portion of the Pt atoms of the Pt_subnano_ form new metallic bonds with Fe atoms and simultaneously undergo chemical bonding with N atoms. It is conceivable that the Pt_subnano_ particles generated in situ were functionalized by both Fe and N atoms and, thus, exhibited unprecedented durability. In contrast, some of the particles grow into very large particles (seen in Figure 4) due to the Ostwald ripening^22^ as shown in Figure 8(B). The contribution of these large particles to the electrocatalytic properties is considered to be negligible.

### 3.6 Change in H_2_O_2_ Yield

**Figure 9:**
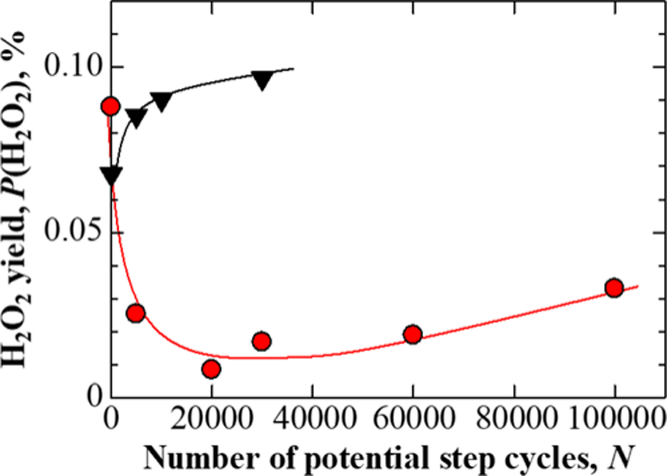
Plots of H_2_O_2_ yields, *P*(H_2_O_2_), at nafion-coated PtCl_*n*_/Fe–N–C (red circle solid) and Pt/CB (▼) electrodes as a function of the number of potential step cycles, *N*. The potential of the working electrode was 0.70 V.

Another advantage of the present Pt_subnano_/Fe–N–C catalyst is a low rate of H_2_O_2_ production as a byproduct of the ORR. The extent of H_2_O_2_ production, *P*(H_2_O_2_), was calculated by the equation
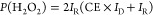
3where *I*_D_ and *I*_R_ are the currents associated with the hydrodynamic voltammetry process and with H_2_O_2_ oxidation at the disk and ring electrodes, respectively (refer to Figure S4), and CE is the collection efficiency for the RRDE system as calculated using eqs S1–S3 in the SI. Figure 9 plots the *P*(H_2_O_2_) values at 0.70 V on Nafion-coated PtCl_*n*_/Fe–N–C and Pt/CB electrodes during the AST. The *P*(H_2_O_2_) values obtained from the Pt/CB electrode evidently increased with *N* as a result of catalyst degradation.^37^ Surprisingly, the *P*(H_2_O_2_) values on the PtCl_*n*_/Fe–N–C rapidly decreased with increases in N, reaching a minimum (0.01%) at 20,000 cycles. It has been reported that the presence of strongly adsorbed species such as sulfonate groups in Nafion can promote the formation of H_2_O_2_ on Pt.^38^ Specifically, sulfonate groups are selectively adsorbed on the Pt(111) planes at 3-fold hollow sites^39−41^ where they block the adsorption of O_2_. As a result, O_2_ molecules are adsorbed in a linear configuration^36,42^ such that H_2_O_2_ production occurs via the two-electron reduction pathway.^43,44^ The H_2_O_2_ yield is therefore affected by the Pt particle size because the proportion of Pt(111) exposed on the surfaces of the Pt particles decreases with decreasing particle size.^45^ In the case of PtCl_*n*_/Fe–N–C, the average Pt particle size of 1.4 nm was decreased to 0.5 nm during AST (as shown in Figure 4). This finding suggests that H_2_O_2_ formation would have been suppressed because the Pt(111) planes were almost completely absent. This decrease in the level of H_2_O_2_ generation will be very helpful in preventing the degradation of the polymer electrolyte membrane and gaskets in PEFCs. Thus, this Pt_subnano_/Fe–N–C catalyst has significant potential with regard to extending the catalyst life along with the life of various fuel cell components.

## 4 Conclusions

Ultrafine Pt particles were formed in situ by the potential cycling of PtCl_*n*_/Fe–N–C in an acidic solution. During potential cycling between 0.6 and 1.0 V (vs RHE), PtCl_*n*_ was reduced to Pt(0) and leached Pt^*n*+^ ions were trapped by nearby Fe and N atoms. This resulted in the growth of subnanometer-sized Pt particles (with diameters of approximately 0.5 nm) each consisting of several atoms (Pt_subnano_). The electrochemical performance of Pt_subnano_/Fe–N–C as a cathode catalyst for PEFCs was evaluated and found to exceed the highest activity yet reported. The ORR mass-based activity was approximately 8 times higher than that of a standard Pt/CB catalyst and the initial performance of this new material was maintained for 100,000 potential cycles. This excellent durability is attributed to the bifunctional interaction of Pt with Fe and N atoms. This stabilization of subnanometer-sized Pt particles represents a breakthrough that could allow platinum consumption to be reduced by minimizing particle sizes. In addition, the bifunctional interactions in this system might lead to new developments in nanoparticle materials other than as catalysts for fuel cells. AST trials of the present Pt_subnano_/Fe–N–C within the operational temperature range for PEFCs are in progress in our laboratory, using 25 cm^2^ single cell and stack formations, and very high durability has been observed. The results will be presented elsewhere.

## Supporting Information Available

Detailed the typical properties of the commercial Pt/CB and *PtCl*_n_*/Fe–N–C catalysts; AST protocol; cyclic voltammograms; hydrodynamic ORR voltammograms; *I*^–1^ vs ω^1/2^ plots and equations used to calculate CE (PDF)
