# Supplementary material for: In Situ Formation of Highly Durable Subnanometer Platinum Particle Electrocatalysts for Polymer Electrolyte Fuel Cells
Source: ACS Omega. 2024 Jun 11;9(25):27499–508. doi: 10.1021/acsomega.4c02723 (PMC11209685; doi:10.1021/acsomega.4c02723)
Supplement: Supplementary file 1 — ao4c02723_si_001.pdf [file ao4c02723_si_001.pdf]

Supporting Information

In situ formation of highly durable sub-nanometre platinum particle electrocatalysts for polymer electrolyte fuel cells

Hiroshi Yano, Kouta Iwasaki

New Field Pioneering Division, Toyota Boshoku Corp. 1-1, Toyoda-cho, Kariya, Aichi, 448-8651 Japan

Table S1 Typical properties of the commercial Pt/CB catalyst and preparation condition of catalysts suspension

	Specific surface area of carbon, *SSA* (m^2 g^{-1})	Pt loaded, *y* (wt %)	Particle size, *d* (nm)	Inter-particle distance, $X_{Pt\text{-}Pt}$ (nm)	Amount of catalyst in the ink suspension, $C_{cat.}$ (g L^{-1})	Amount of the pipetting the suspension, V_{ink} (μL)	Amount of Pt attached in the catalyst layer, m_{Pt} (μg cm^{-2})	Amount of carbon attached in the catalyst layer, m_c (μg cm^{-2})
Pt/CB (commercial)	800	46.6	2.5±0.4	13.2	1.0	4	14.8	17.0

	Specific surface area of carbon, SSA ($m^2\ g^{-1}$)	Pt loaded, y (wt %)	Particle size, d (nm)	Inter-particle distance, $X_{Pt\text{-}Pt}$ (nm)	Amount of catalyst in the ink suspension, $C_{cat.}$ ($g\ L^{-1}$)	Amount of the pipetting the suspension, V_{ink} (μL)	Amount of Pt attached in the catalyst layer, m_{Pt} ($\mu g\ cm^{-2}$)	Amount of carbon attached in the catalyst layer, m_c ($\mu g\ cm^{-2}$)
*$PtCl_n$/Fe-N-C	560	26.7	1.3±0.1	6.6	1.0	18	38.2	105

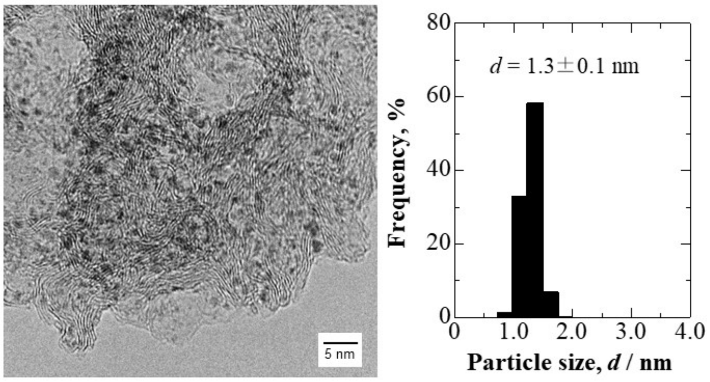

Figure S1 Typical properties, TEM image, and particle size distribution histogram of the *$PtCl_n$/Fe-N-C catalyst.

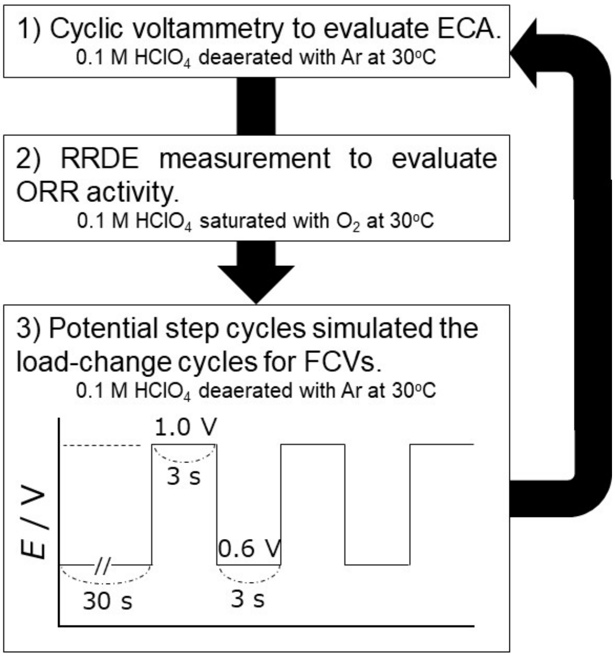

Figure S2 The accelerated durability testing procedure used to evaluate degradation of the $PtCl_n$/Fe-N-C, $PtCl_n$/GCB, $PtCl_n$/MPC and Pt/CB catalysts.

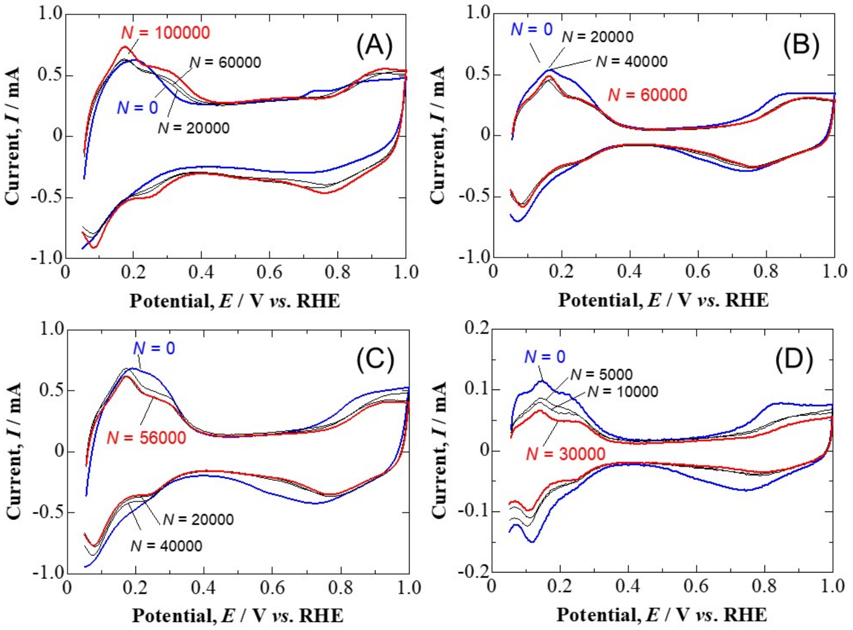

Figure S3 Cyclic voltammograms employed to determine *ECA* values for Nafion-coated (A) $PtCl_n$/Fe-N-C, (B) $PtCl_n$/GCB, (C) $PtCl_n$/MPC and (D) Pt/CB electrodes in 0.1 M $HClO_4$ solutions deaerated with Ar at 30 ˚C at a scan rate of 50 mV s^{-1}.

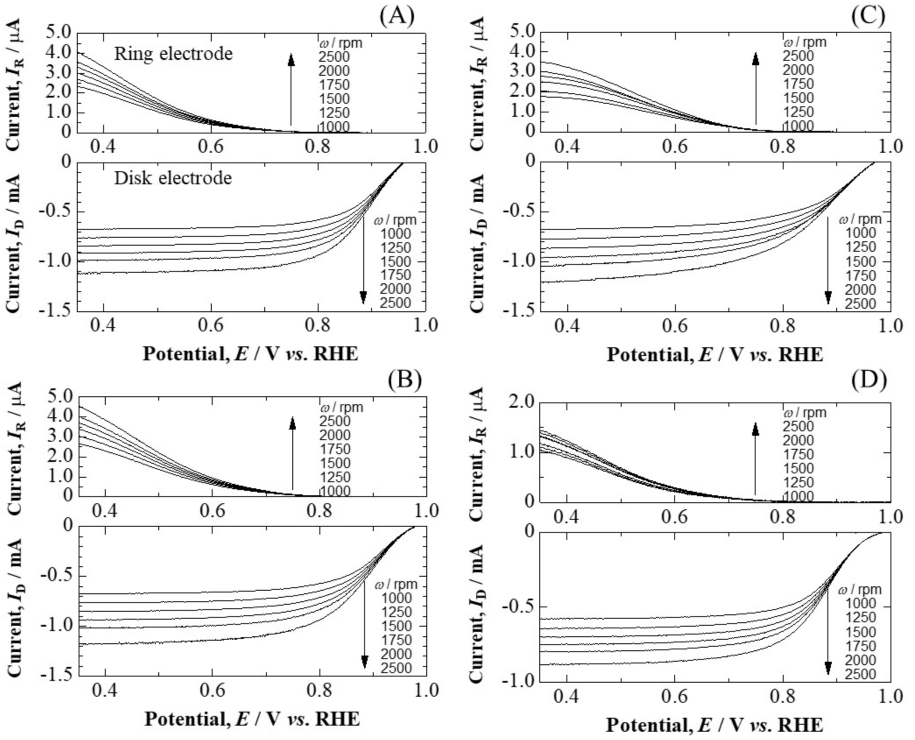

Figure S4 Hydrodynamic voltammograms for the ORR in O_2-saturated 0.1 M $HClO_4$ solution at a Nafion-coated (A) PtCl$_n$/Fe-N-C, (B) PtCl$_n$ /GCB, (C) PtCl$_n$ /MPC, and (D) Pt/CB electrodes at 30˚C and simultaneously acquired currents at a Pt ring electrodes for the oxidation of H_2O_2. Potential scan rate of the disk electrode = 10 mV s^{-1}; rotation rate, from 1000 to 2500 rpm; potential of the ring electrode = 1.2 V.

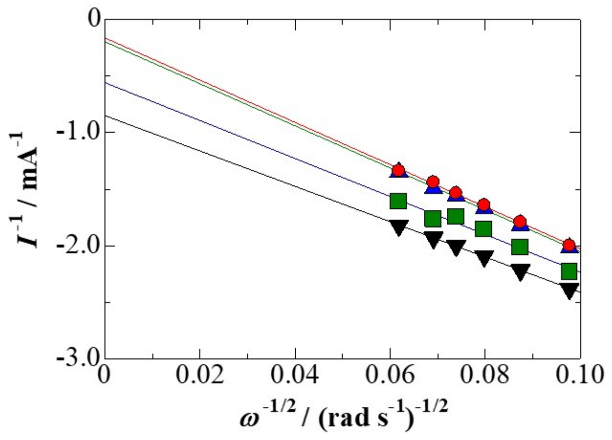

Figure S5 Koutecky-Levich plots obtained from hydrodynamic voltammograms for the ORR with Nafion-coated $PtCl_n$/Fe-N-C (●), $PtCl_n$/GCB (▲), $PtCl_n$/MPC (■) and Pt/CB (▼) electrodes in 0.1 M $HClO_4$ solutions at 30 ˚C. Potential of working electrode = 0.85 V *vs*. RHE.

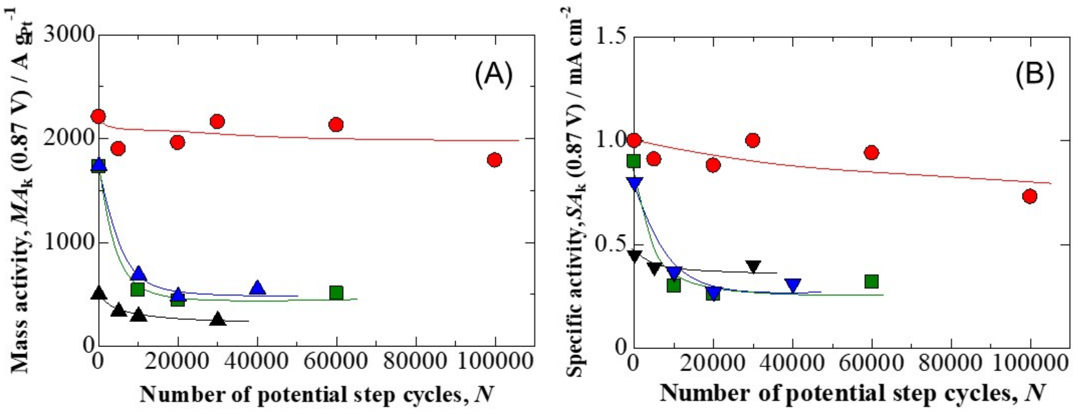

Figure S6 Change in (A) kinetically-controlled mass activity, MA_k, and (B) kinetically-controlled area-specific activity, SA_k, at Nafion-coated $PtCl_n$/Fe-N-C (●), $PtCl_n$/GCB (▲), $PtCl_n$/MPC (■), and Pt/CB (▼) electrodes as a function of the number of potential step cycles, N. The values of SA_k and MA_k were evaluated at 0.87 V vs. RHE.

Collection efficiency calculations

The extent of H_2O_2 production, $P(H_2O_2)$, was calculated as

$$P(H_2O_2) = 2I_R(CE \times I_D + I_R),$$

where I_D and I_R are the currents at the disk and ring electrodes, respectively, and CE is the collection efficiency for the RRDE system as calculated from the relationships

$$CE = 1 - F\left(\frac{\alpha}{\beta}\right) + \beta^{2/3}[1 - F(\alpha)] - (1 + \alpha + \beta)^{2/3}\left\{1 - F\left[\left(\frac{\alpha}{\beta}\right)(1 + \alpha + \beta)\right]\right\}, \quad \text{(S1)}$$

$$\alpha = \left(\frac{r_1}{r_2}\right)^3 - 1, \quad \beta = \left(\frac{r_3}{r_1}\right)^3 - \left(\frac{r_2}{r_1}\right)^3, \quad \text{(S2)}$$

and

$$F(\theta) = \frac{\sqrt{3}}{4\pi} ln \frac{\left(1+\theta^{1/3}\right)^3}{1+\theta} + \frac{3}{2\pi}\arctan\left(\frac{2\theta^{1/3}-1}{3^{1/2}}\right) + 4 \ . \quad \text{(S3)}$$

Here, r_1, r_2 and r_3 are the disk electrode radius, the inner radius of the ring electrode and the outer radius of the ring electrode, respectively.
